# *N*-Acetylcysteine Reduces ROS-Mediated Oxidative DNA Damage and PI3K/Akt Pathway Activation Induced by *Helicobacter pylori* Infection

**DOI:** 10.1155/2018/1874985

**Published:** 2018-04-26

**Authors:** Chuan Xie, Jian Yi, Jing Lu, Muwen Nie, Meifang Huang, Jianfang Rong, Zhenhua Zhu, Jiang Chen, Xiaoliang Zhou, Bimin Li, Haiming Chen, Nonghua Lu, Xu Shu

**Affiliations:** ^1^Department of Gastroenterology, The First Affiliated Hospital of Nanchang University, Nanchang, Jiangxi 330006, China; ^2^Department of Gastroenterology, Yichun People's Hospital, Yichun, Jiangxi 336000, China; ^3^Department of Rheumatology, Ganzhou People's Hospital, Ganzhou, Jiangxi 341000, China; ^4^Queen Mary Institute, Nanchang University, Nanchang, Jiangxi 330000, China; ^5^Department of Gastroenterology, Shangrao People's Hospital, Ganzhou, Jiangxi 334000, China

## Abstract

**Background:**

*H. pylori* infection induces reactive oxygen species- (ROS-) related DNA damage and activates the PI3K/Akt pathway in gastric epithelial cells. *N*-Acetylcysteine (NAC) is known as an inhibitor of ROS; the role of NAC in *H. pylori*-related diseases is unclear.

**Aim:**

The aim of this study was to evaluate the role of ROS and the protective role of NAC in the pathogenesis of *H. pylori*-related diseases.

**Method:**

An *in vitro* coculture system and an *in vivo* Balb/c mouse model of *H. pylori*-infected gastric epithelial cells were established. The effects of *H. pylori* infection on DNA damage and ROS were assessed by the comet assay and fluorescent dichlorofluorescein assay. The level of PI3K/Akt pathway-related proteins was evaluated by Western blotting. The protective role of *N*-acetylcysteine (NAC) was also evaluated with *in vitro* and *in vivo H. pylori* infection models.

**Results:**

The results revealed that, *in vitro* and *in vivo*, *H. pylori* infection increased the ROS level and induced DNA damage in gastric epithelial cells. NAC treatment effectively reduced the ROS level and inhibited DNA damage in GES-1 cells and the gastric mucosa of Balb/c mice. *H. pylori* infection induced ROS-mediated PI3K/Akt pathway activation, and NAC treatment inhibited this effect. However, the gastric mucosa pathological score of the NAC-treated group was not significantly different from that of the untreated group. Furthermore, chronic *H. pylori* infection decreased APE-1 expression in the gastric mucosa of Balb/c mice.

**Conclusions:**

An increased ROS level is a critical mechanism in *H. pylori* pathogenesis, and NAC may be beneficial for the treatment of *H. pylori*-related gastric diseases linked to oxidative DNA damage.

## 1. Introduction

Gastric cancer is a common malignant tumor worldwide. The latest epidemiological data indicates that worldwide, gastric cancer is the fourth leading cause of new cancer cases in males and fifth in females and is the third leading cause of cancer-related death in males and fifth in females [1]. At diagnosis, most new gastric cancer cases are already in advanced stages, with limited treatment and poor prognosis [1]. Since the underlying mechanisms responsible for the development of gastric cancer are still poorly understood, further studies are needed to develop novel preventative strategies. However, *Helicobacter pylori* (*H. pylori*) infection, which has been classified as a class I carcinogen for gastric cancer by the International Agency for Research of Cancer, induces chronic nonatrophic gastritis that can progress into intestinal metaplasia, dysplasia, and, ultimately, gastric cancer [2, 3].

Our previous studies have shown that *H. pylori* infection induces DNA double-strand breaks (DSBs) *in vivo* [4], which is the most serious type of DNA damage. Direct contact between the host and pathogen may contribute to *H. pylori*-induced DSBs [5]. More importantly, oxidative stress-induced DNA damage may be an important factor in the pathogenesis of *H. pylori*-associated gastric diseases [6]. *H. pylori* activates NADPH oxidase and increases the production of reactive oxygen species (ROS) in gastric epithelial cells, independent of inflammatory cells [7, 8]. ROS production by *H. pylori* activates the NF-*κ*B pathway and plays an important role in apoptosis and DNA damage in gastric epithelial cells [6]. In AGS cells, lycopene, a natural antioxidant, inhibits the increase in ROS levels, apoptosis, and DNA damage induced by *H. pylori* infection [9].

PI3K/Akt pathway signaling is essential for maintaining the integrity of fundamental cellular processes, cell growth, survival, death, and metabolism. Hyperactivation of PI3K/Akt signaling has been reported in many types of human cancers; thus, targeting the regulators in this pathway has attractive therapeutic potential [10, 11]. It is activated by extracellular signals and is downregulated by phosphatase and tensin homolog (PTEN). Our previous studies revealed that PI3K/Akt pathway activation participates in gastric carcinogenesis [12]. During the early stage of gastric lesions, *H. pylori* infection activates the PI3K/Akt pathway and promotes cell survival via increased PTEN phosphorylation at residues Ser380/Thr382/383 [12]. According to previous research, ROS not only activates PI3K directly to amplify its downstream signaling but also concurrently inactivates PTEN, which negatively regulates Akt activation [13]. In addition, by stimulating oxidative metabolism, AKT promotes mitochondrial oxygen consumption and promotes ROS accumulation [14]. NAC is known as an inhibitor of ROS and an antioxidant. However, whether NAC has a protective role in *H. pylori*-related diseases is unclear. Therefore, the aims of this study were to investigate the effect of NAC on gastric epithelial cell with *H. pylori* and to determine the role of ROS and the PI3K/Akt pathway in *H. pylori*-induced oxidative DNA damage in gastric epithelial cells, which may help to discover a new strategy against *H. pylori*-related diseases.

## 2. Materials and Methods

### 2.1. Cell Lines and *H. pylori* Infection

The immortalized human gastric epithelial cell line GES-1 was cultured in Dulbecco's modified Eagle's medium (DMEM) supplemented with 10% fetal bovine serum, 100 U penicillin, and 100 *μ*g/ml streptomycin (Gibco of Thermo Fisher Scientific Inc., Waltham, MA, USA) at 37°C in an atmosphere of 5% CO_2_. The *H. pylori* strain (ATCC43504, CagA^+^, and VacA^+^) was cultured on Campylobacter agar plates containing 10% sheep serum and was incubated at 37°C under microaerophilic conditions for 24 h. The bacteria were suspended in DMEM or Brucella broth, and bacterial density was estimated by spectrophotometry (A660). GES-1 cells were cultured in the presence of *H. pylori* at different multiplicities of infection (MOIs) as described previously [12].

### 2.2. Reagents

Pharmacological inhibition of PI3K was achieved with LY294002 (40 *μ*M; Sigma-Aldrich, St. Louis, MO, USA). Free radical production was inhibited with *N*-acetyl-L-cysteine (NAC) (200 mg; Sigma-Aldrich, St. Louis, MO, USA).

### 2.3. Balb/c Mice

Sixty six- to eight-week-old clean-grade male Balb/c mice (30–50 g) were provided by Hunan Slac Jingda Experimental Animal Co. Ltd. (Changsha, Hunan, China) and were maintained in an isolated clean room with regulated temperature (20–22°C), humidity (approximately 55%), and a 12/12 h light/dark cycle with ad libitum access to chow and water. After one week of observation, the Balb/c mice were divided into five groups (groups A–E, *n* = 12 for each group). Group A was given 1 ml orogastric infusions of sterile Brucella broth for 14 days, followed by distilled water. Group B was given 1 ml orogastric infusions of 1 × 10^9^ colony-forming units of *H. pylori*-type strain SS1 (CagA^+^ and VacA^+^) once every two days for a total of seven infusions, followed by distilled water. Group C was given 1 ml orogastric infusions of sterile Brucella broth for 14 days, followed by NAC (6 mg/ml, 0.5 ml/20 g) infusions once a day until the animals were sacrificed. Group D was given 1 ml orogastric infusions of 1 × 10^9^ colony-forming units of *H. pylori*-type strain SS1 once every two days for a total of seven infusions, followed by NAC once a day for 12 weeks, and then the mice received distilled water. Group E was given 1 ml orogastric infusions of 1 × 10^9^ colony-forming units of *H. pylori*-type strain SS1 once every two days for a total of seven infusions, followed by distilled water for 12 weeks; then, the mice were given NAC until the time of sacrifice. Each group was euthanized at 24 weeks, and linear strips of gastric tissue, extending from the squamocolumnar junction through the proximal duodenum, were collected. Hematoxylin and eosin (HE) staining was performed. Two pathologists, blinded to the group data, reviewed the biopsies and discussed the pathological score according to the updated Sydney System [15]. All animal experiments and procedures were approved by the Ethics Committee of The First Affiliated Hospital of Nanchang University.

### 2.4. Immunoblotting

Western blotting was performed according to standard methods, as described previously [16], using the following antibodies: anti-Akt (#4691; 1 : 1000), anti-p-Akt (Ser473) (#9271; 1 : 1000), anti-GSK-3*β* (#12456; 1 : 1000), anti-p-GSK-3*β* (Ser9) (#9323; 1 : 1000) (Cell Signaling Technology, Danvers, MA, USA), anti-APE1 (ab189474; 1 : 1000) (Abcam), and anti-*β*-actin (sc-1615-R; 1 : 1000; Santa Cruz Biotechnology, Dallas, TX, USA).

### 2.5. Immunohistochemistry

Immunohistochemistry was performed on paraffin sections of human biopsy specimens or Mongolian gerbil gastric tissues using anti-Akt (ab8805; 1 : 400), anti-p-Akt (Ser473) (ab66138; 1 : 400), anti-GSK-3*β* (ab32391; 1 : 400), anti-p-GSK-3*β* (Ser9) (ab75814; 1 : 500), anti-APE1 (ab189474; 1 : 400), and anti-8-OHdG (ab62623; 1 : 400) (Abcam, Cambridge, UK) antibodies following previously described methods [16, 17]. The stained sections were reviewed and scored from five randomly selected high-power fields (40x objective lens) by two pathologists blinded to the histopathological data. To obtain the final score, grading discrepancies were re-reviewed and discussed. Epithelial cells with yellow or brown staining in the nucleus and/or cytoplasm were defined as positive for immunoreactivity. In each field, out of 100 cells, the percentage that were immunoreactive was averaged from the five fields and was scored as follows: 0 = <5.0% immunoreactive; 1 = 5.1–25.0%, 2 = 25.1–50.0%, 3 = 50.1–75.0%, and 4 = >75.0%. Moreover, the staining intensity was also semiquantitatively assessed as follows: 0 = no staining, 1 = weak staining, 2 = moderate staining, and 3 = strong staining. The overall protein expression level was reported as a grade, calculated from the integral score of the “area × intensity” as follows: grade  1 = score  0–2 (negative), grade  2 = score  3–5 (weakly positive), grade  3 = score  6–8 (moderately positive), and grade  4 = score  9–12 (strongly positive).

### 2.6. Comet Assay

DNA damage was evaluated by comet assay in the GES-1 cell line. Cells were resuspended in 0.6% low-melting-point agarose and then were transferred to a glass microscope slide, precoated with a layer of 0.7% agarose. The slides were incubated in lysis buffer (2.5 M NaCl, 100 mM Na_2_ EDTA, 1% *N*-lauroylsarcosine, 10 mM Tris, NaOH to pH 10.0, and 1% Triton X-100) at 4°C for 1 h in a light-resistant container and then were electrophoresed at 30 V for 30 min. The slides were neutralized with 0.4 mM Tris-HCl (pH = 7.5) for 20 min. Comet tails were stained with propidium iodide (PI) and were analyzed by a fluorescence microscope. For each treatment, over 70 cells were analyzed for the comet tail moment using CASP 6.0 with the comet assay.

### 2.7. Measurement of Intracellular ROS and MDA Content

ROS levels in GES-1 cells or the gastric mucosa of Balb/c mice were measured using a ROS assay kit (C1300, Applygen Technologies, Beijing, China). In the Balb/c mouse gastric mucosa, malondialdehyde (MDA) levels were determined using an MDA assay kit (A003-1, Nanjing Jiancheng Bioengineering Institute, China). The fluorescent probe dichlorofluorescein (DCF) was measured (excitation at 488 nm and emission at 525 nm) with a Bio-Rad 680 multilabel counter (Bio-Rad Laboratories, CA, USA) or a Live Cell Imaging System (IX81, Olympus Corporation, Japan).

### 2.8. Statistical Analysis

Data are presented as the means ± standard deviation (SD) or a percentage of the control. The chi-square test was performed to evaluate differences in categorical variables, such as gender, among the various defined groups. One-way analysis of variance (ANOVA) was used to determine differences in the numerical variables, such as patient age, among the groups. The Kruskal-Wallis or Mann–Whitney test was used to determine differences in the numerical variables among differently defined groups. A *p* value of < 0.05 was considered statistically significant.

## 3. Results

### 3.1. *H. pylori* Induces DNA Damage and Increases the ROS Level in GES-1 Cells, but NAC Pretreatment Protects Cells from Oxidative Damage

**Figure 1:**
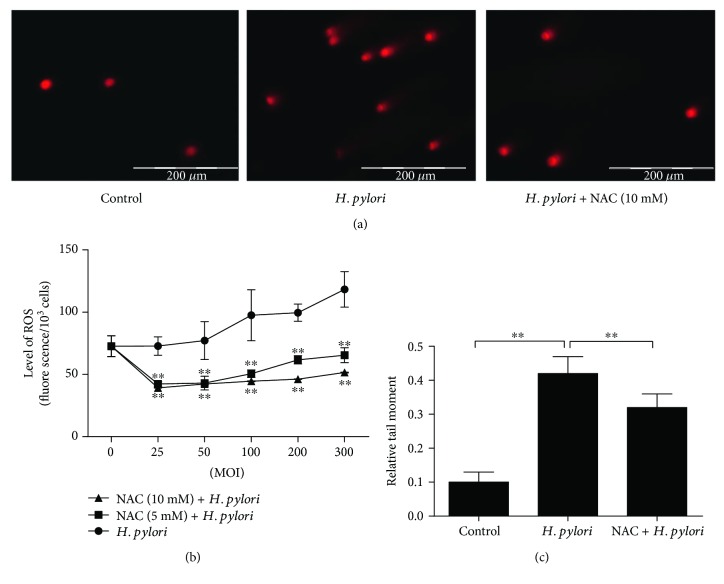
*H. pylori* induces DNA damage and increases the ROS level in gastric epithelial cells (GES-1) cells, but pretreatment with NAC protects cells from oxidative damage. (a) At 6 h of culture, DNA damage was examined using comet formation assay, scale bar = 200 *μ*m. (b) ROS levels were determined by measuring the level of fluorescent DCF; ^∗∗^*p* < 0.01 compared to the control group. (c) Comet formation was quantitatively assessed according to the relative tail moment at 6 h of culture; ^∗∗^*p* < 0.01.

Incubating nonmalignant GES-1 cells with *H. pylori* for 6 h increased the ROS level, which was positively correlated with the *H. pylori* MOI. NAC is an antioxidant that can inhibit the generation of ROS. To further assess whether NAC inhibits the *H. pylori*-induced increase in ROS levels, we pretreated GES-1 cells with NAC (5 and 10 mM) for 1 h; then, the cells were cocultured with *H. pylori*. Compared to the *H. pylori* group, both NAC-treated groups had reduced ROS levels; the 10 mM NAC treatment was more effective but was not significantly different from the 5 mM treatment (Figure 1(b)).

In addition, the neutral comet assay was performed to evaluate the DNA damage in GES-1 cells after coculture with *H. pylori* (MOI = 300). After incubating for 6 h, the *H. pylori*-cocultured cells presented with a significant upregulation in the number of comet cells with increased tail moments. However, compared to the untreated cells, the GES-1 cells pretreated with 10 mM NAC had less *H. pylori*-induced DNA damage (Figures 1(a) and 1(c)).

### 3.2. ROS Regulates the PI3K/Akt Pathway in GES-1 Cells, Which Is Activated by *H. pylori* Infection

**Figure 2:**
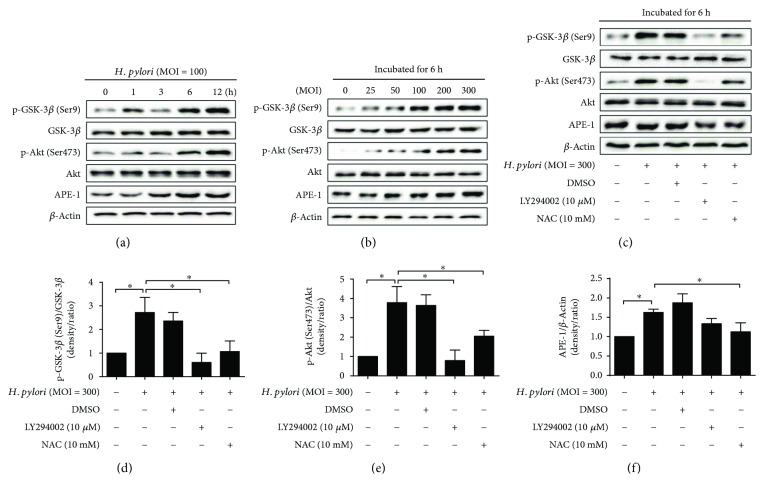
ROS regulates the PI3K/Akt pathway, which is activated by *H. pylori* infection. (a) Gastric epithelial cells (GES-1) were incubated with *H. pylori* (multiplicity of infection (MOI) = 100) for various lengths of time. Immunoblots of GSK-3*β*, p-GSK-3*β* (Ser9), Akt, p-Akt (Ser473), APE-1, and beta-actin expression. (b) GES-1 cultures were incubated for 6 h at various *H. pylori* MOI. (c–f) GES-1 cultures were pretreated with LY294002 or NAC and then were incubated for 6 h with *H. pylori* (MOI = 300). Representative immunoblots and quantification of the above protein levels; ^∗^*p* < 0.05.

*H. pylori* has been reported to activate the PI3K/Akt pathway, but whether this activation effect is concentration- or time-dependent is unclear. As expected, Akt and GSK-3*β* phosphorylation was significantly increased following a 6 h incubation with *H. pylori* (MOI = 100 for 0, 1, 3, 6, and 12 h), and this increase was time-dependent; however, the total Akt and GSK-3*β* levels were not changed by *H. pylori* infection. Similarly, a significant concentration-dependent increase in Akt and GSK-3*β* phosphorylation was also observed in GES-1 cells that had been incubated with *H. pylori* (MOI = 0, 25, 50, 100, 200, and 300 for 6 h) (Figures 2(a)–2(f)).

APE-1 is a limiting enzyme in DNA damage repair. It has been reported that ROS regulates the expression of APE-1, which is an indicator of oxidative stress. In our *in vitro* study, *H. pylori* infection increased the expression of APE-1 in a time- and concentration-dependent manner (Figures 2(a) and 2(b)).

To investigate whether the *H. pylori*-induced activation of the PI3K/Akt pathway is linked to increased ROS levels, we treated *H. pylori*-infected (MOI = 300) cells with 10 *μ*M LY294002 (a PI3K inhibitor) and 10 mM NAC. After 6 h of culturing, *H. pylori*-induced PI3K/Akt pathway activation was almost completely inhibited by LY294002 treatment. Similarly, NAC pretreatment also downregulated Akt and GSK-3*β* phosphorylation. Pretreatment with NAC also decreased the overexpression of APE-1 induced by *H. pylori* (Figures 2(c)–2(f)).

### 3.3. *H. pylori* Infection Induces Oxidative DNA Damage and Activates the PI3K/Akt Pathway in Balb/c Mouse Gastric Tissue, and NAC Alleviates These Effects

**Figure 3:**
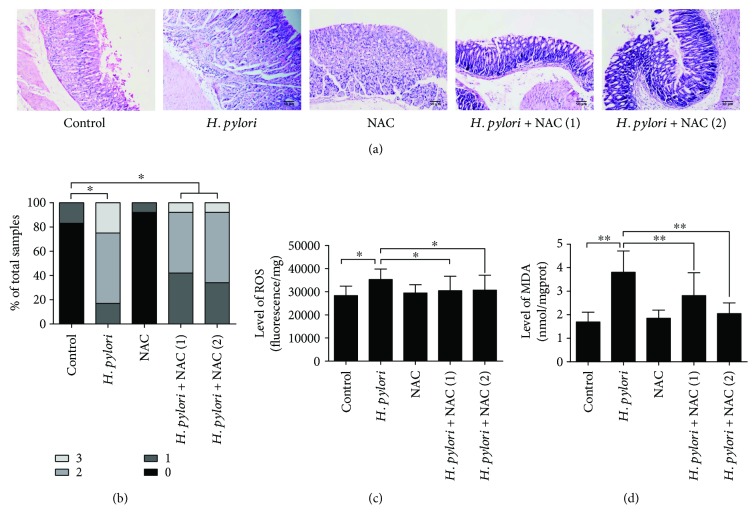
*H. pylori* infection induces gastritis and oxidative damage in the gastric tissue of Balb/c mice. Gastric tissue sections from *H. pylori-*infected mice were taken after 24 weeks of infection. (a) Pathological changes were evaluated by HE staining. (b) Gastritis histopathology was graded according to the updated Sydney System. (c) Gastric ROS levels were determined by measuring DCF fluorescence. (d) MDA levels in the gastric mucosa were detected. Scale bar = 50 *μ*m; ^∗^*p* < 0.05 and ^∗∗^*p* < 0.01.

To further verify the *in vitro* results above, we performed *in vivo* studies. Balb/c mice were successfully infected with *H. pylori*, which was confirmed by pathological detection; none of the animals challenged with Brucella broth alone presented with any detectable evidence of *H. pylori* infection (data not shown). In the *H. pylori*-infected animals, moderate to severe gastritis was accompanied by polymorphonuclear neutrophil infiltration in the mucosa and submucosa after 24 weeks of infection. The gastritis histopathological score was not significantly altered after the NAC intervention (groups D and E) (Figures 3(a) and 3(b)).

**Figure 4:**
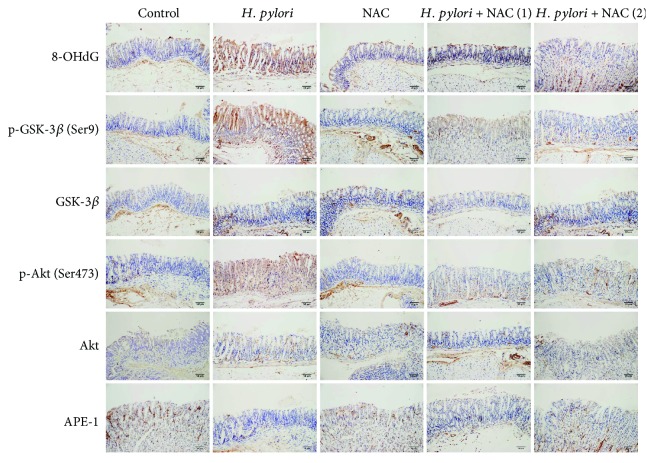
*H. pylori* infection induces DNA damage and activates the PI3K/Akt pathway *in vivo*. Gastric tissue samples were stained with antibodies against 8-OHdG, GSK-3*β*, p-GSK-3*β* (Ser9), Akt, p-Akt (Ser473), and APE-1. Scale bar = 50 *μ*m.

To confirm that *H. pylori* infection induces oxidative DNA damage *in vivo*, 8-oxo-2′-deoxyguanosine (8-OHdG; a metabolic product of DNA damage) was immunohistochemically analyzed, and ROS, MDA, and reduced glutathione (GSH) levels were measured in the gastric tissue samples of Balb/c mice. The expression of 8-OHdG was increased, and the ROS and MDA levels were higher after 24 weeks of *H. pylori* infection. NAC treatment (groups D and E) alleviated the oxidative DNA damage caused by *H. pylori* infection (Figure 4). Similarly, compared to the group without NAC treatment, the NAC-treated group presented with reduced ROS and MDA levels (Figures 3(c) and 3(d)).

**Figure 5:**
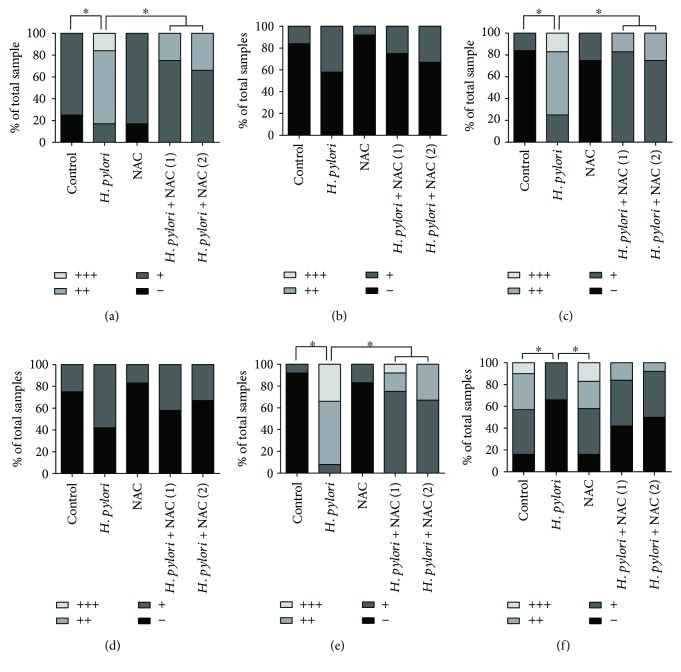
Immunoreactive cells were semiquantitatively assessed. The protein expression levels are expressed as grades 1–4. The proportion of each grade is shown. (a) 8-OHdG; (b) Akt; (c) p-Akt (Ser473); (d) GSK-3*β*; (e) p-GSK-3*β* (Ser9); (f) APE-1; ^∗^*p* < 0.05.

**Figure 6:**
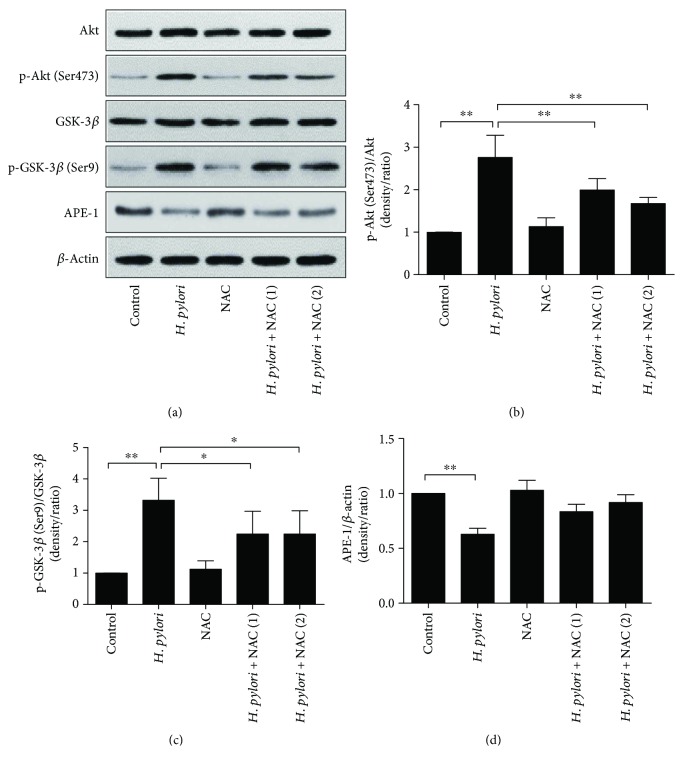
Immunoblots of PI3K/Akt pathway-related proteins in the gastric mucosa. Immunoblots of (a) GSK-3*β*, p-GSK-3*β* (Ser9), Akt, p-Akt (Ser473), APE-1, and APE-actin; (b) quantification of the relative protein expression levels (expressed as fold of control); ^∗^*p* < 0.05 and ^∗∗^*p* < 0.01.

To investigate whether *H. pylori* infection activated the PI3K/Akt pathway in the gastric tissue of Balb/c mice, the expression of the PI3K/Akt pathway-related proteins Akt, p-Akt (Ser473), GSK-3*β*, and p-GSK-3*β* (Ser9) was evaluated by immunohistochemistry and immunoblotting. At 24 weeks after *H. pylori* infection, the p-Akt (Ser473) and p-GSK-3*β* (Ser9) levels were increased, but NAC treatment inhibited this increase and reduced the level of oxidative DNA damage (Figures 4–6). In contrast to the *in vitro* study, we observed that *H. pylori* infection inhibited the expression level of APE-1 in the gastric tissue of the Balb/c mice, and NAC treatment was unable to restore APE-1 expression (Figures 4–6).

## 4. Discussion

A previous study proved that *H. pylori* infection induces oxidative DNA damage and apoptosis in gastric epithelial cells [9]. This study has further demonstrated that *H. pylori*-induced DNA damage is connected to the ROS level. In addition, the antioxidant NAC alleviated the DNA damage induced by *H. pylori* by decreasing the ROS level.

The PI3K/Akt pathway is frequently activated in gastric carcinogenesis and vital to gastric cancer development [18]. This pathway was found to be activated by *H. pylori* infection both *in vivo* and *in vitro*, which is consistent with previous studies [17, 19–22]. Our previous study revealed that *H. pylori* increases PTEN phosphorylation at residues Ser380/Thr382/383, which activates the PI3K/Akt pathway and promotes cell survival [12]. However, reduced survival and increased proliferation have been previously reported in *H. pylori*-infected cells [23]. These seemingly contradictory results reveal the diverse effects of *H. pylori* infection and may be due to variations in different experimental models [23]. We speculated that these results may be explained by the dual role of ROS. Moderate ROS levels have been proven to be required for proper stem cell differentiation and renewal via signaling pathway activation. The activation of cellular responses due to slight increases in ROS levels can increase signaling pathways that counter the normal aging process. However, high ROS levels can hyperactivate signaling pathways that promote inflammation, cancer, and cell death, leading to an accelerated aging phenotype [24]. Bae et al. reported that *H. pylori* infection induces the oxidative DNA damage response, cell cycle arrest, and apoptosis in the gastric mucosa of Balb/c mice [25]. Our study shows that a high MOI of *H. pylori* increases high levels of ROS and then induces oxidative DNA damage and neutrophil infiltration, which may develop into acute gastritis or peptic ulcers. However, low concentrations of *H. pylori* infection slightly increase the ROS level, leading to persistent activation of the PI3K/Akt pathway, resulting in activation of p-Akt (Ser473) and downstream protein p-GSK-3*β* (Ser9), which may contribute to cell proliferation and gastric carcinogenesis. This study also shows that NAC decreases the level of ROS and inhibits PI3K/Akt pathway activation.

NAC is a precursor of L-cysteine that results in glutathione elevation biosynthesis. NAC stimulates glutathione biosynthesis, promotes detoxification, and acts directly as a scavenger of free radicals. It is a powerful antioxidant and a potential treatment option for diseases characterized by the generation of free oxygen radicals [26]. It is also recommended as a potential treatment option for different disorders which resulted from the generation of free oxygen radicals. Additionally, it is a protected and endured mucolytic drug that mellows tenacious mucous discharges. It has been used for treatment of various diseases in a direct action or in a combination with some other medications [27]. However, the effect of NAC on *H. pylori*-related diseases was rarely researched. NAC administration may exert a beneficial effect on the reduction of *H. pylori* colonization and prevents the development of severe inflammation [28]. Moreover, when used with a three-drug regimen, NAC has an additive effect on the eradication rates of *H. pylori* and appears to be a promising treatment for *H. pylori* infections [29]. In this study, after *H. pylori* infection, both concentrations of NAC reduced the levels of ROS and oxidative injury in the gastric mucosa of Balb/c mice. The protective role of NAC may be attributed to a decrease in generation of ROS. Meanwhile, NAC activity may also relate to increased GSH synthesis and consequent modulation of the oxidative status of gastric cells. However, NAC did not reduce the pathological score; we speculate that the changes in ROS levels preceded the reduction in inflammation. Under a prolonged observation time, pathological changes may be observed.

APE-1 is a master regulator of the cellular response to oxidative stress and is involved in the transcriptional regulation of gene expression during the adaptive cellular response to oxidative stress and in the base excision repair pathway [30]. Interestingly, different *in vitro* and *in vivo* results were observed. In a time-dependent manner, *H. pylori* infection increased the expression of APE-1 in GES-1 cells. However, *H. pylori* infection inhibited the APE-1 level in Balb/c mouse gastric tissue after 24 weeks of infection. These contradictory results are not well understood. We speculate that *H. pylori* infection induces oxidative DNA damage, activating the DNA damage response pathway in gastric epithelial cells. The upregulated expression of APE-1 is conducive for repairing DNA damage. However, chronic *H. pylori* infection may inhibit the expression of APE-1, ultimately leading to genomic instability.

In conclusion, this study indicated that *H. pylori* infection increases the ROS level and induces oxidative DNA damage in gastric epithelial cells. NAC treatment may be beneficial for treating *H. pylori*-related gastric diseases that are linked to oxidative DNA damage. NAC inhibited *H. pylori*-induced DNA damage probably by reducing the ROS levels and thereby suppressing the ROS-induced activation of the PI3K/Akt pathway.

## Abbreviations

*H. pylori*: *Helicobacter pylori*
PI3K: Phosphatidylinositol-4,5-bisphosphate 3-kinase
ROS: Reactive oxygen species
MOI: Multiplicity of infection
NAC: *N*-Acetylcysteine.
