# Correction to “*N*‐Acetylcysteine Reduces ROS‐Mediated Oxidative DNA Damage and PI3K/Akt Pathway Activation Induced by *Helicobacter pylori* Infection”

**DOI:** 10.1155/omcl/9794537

**Published:** 2025-12-16

C. Xie, J. Yi, J. Lu, M. Nie, M. Huang, J. Rong, Z. Zhu, J. Chen, X. Zhou, B. Li, H. Chen, N. Lu, and X. Shu, “*N*‐Acetylcysteine Reduces ROS‐Mediated Oxidative DNA Damage and PI3K/Akt Pathway Activation Induced by *Helicobacter pylori* Infection,” *Oxidative Medicine and Cellular Longevity*, (2018): 1874985, https://doi.org/10.1155/2018/1874985.

In the article, there is an error in Figure 4. Specifically, there is a duplication between representative images of GSK‐3β in the *H. pylori* group and the *H. pylori* + NAC(2) group.

The correct Figure 4 is shown below:

**Figure 4:**
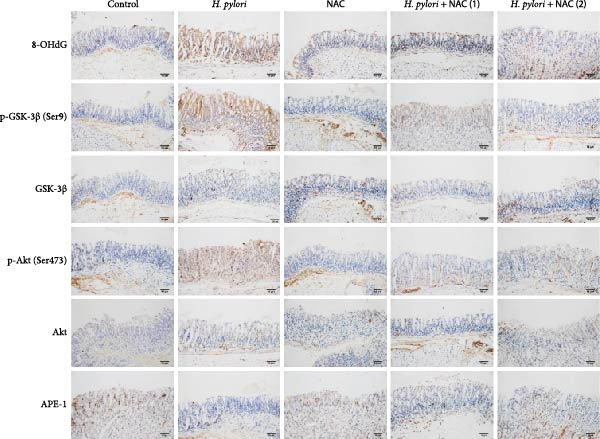
*Helicobacter pylori* infection induces DNA damage and activates the PI3K/Akt pathway in vivo. Gastric tissue samples were stained with antibodies against 8‐OHdG, GSK‐3β, p‐GSK‐3β (Ser9), Akt, p‐Akt (Ser473), and APE‐1. Scale bar = 50 µm.

We apologize for this error.

